# A 4-gene-based hypoxia signature is associated with tumor immune microenvironment and predicts the prognosis of pancreatic cancer patients

**DOI:** 10.1186/s12957-021-02204-7

**Published:** 2021-04-17

**Authors:** Jianfeng Ding, Xiaobo He, Xiao Cheng, Guodong Cao, Bo Chen, Sihan Chen, Maoming Xiong

**Affiliations:** 1 Department of General Surgery, Chaohu Hospital of Anhui Medical University, Chaohu, 238000 Anhui China; 2 Department of General Surgery, The First Affiliated Hospital of Anhui Medical University, Hefei, 230022 Anhui China; 3 Department of Pathology, School of Basic Medicine, Anhui Medical University, Hefei, 230032 Anhui China

**Keywords:** Hypoxia, Hypoxia risk model, Pancreatic cancer, Tumor microenvironment, Immune response, Prognosis

## Abstract

**Background:**

Pancreatic cancer (PAC) is one of the most devastating cancer types with an extremely poor prognosis, characterized by a hypoxic microenvironment and resistance to most therapeutic drugs. Hypoxia has been found to be one of the factors contributing to chemoresistance in PAC, but also a major driver of the formation of the tumor immunosuppressive microenvironment. However, the method to identify the degree of hypoxia in the tumor microenvironment (TME) is incompletely understood.

**Methods:**

The mRNA expression profiles and corresponding clinicopathological information of PAC patients were downloaded from The Cancer Genome Atlas (TCGA) and Gene Expression Omnibus (GEO) database, respectively. To further explore the effect of hypoxia on the prognosis of patients with PAC as well as the tumor immune microenvironment, we established a hypoxia risk model and divided it into high- and low-risk groups in line with the hypoxia risk score.

**Results:**

We established a hypoxia risk model according to four hypoxia-related genes, which could be used to demonstrate the immune microenvironment in PAC and predict prognosis. Moreover, the hypoxia risk score can act as an independent prognostic factor in PAC, and a higher hypoxia risk score was correlated with poorer prognosis in patients as well as the immunosuppressive microenvironment of the tumor.

**Conclusions:**

In summary, we established and validated a hypoxia risk model that can be considered as an independent prognostic indicator and reflected the immune microenvironment of PAC, suggesting the feasibility of hypoxia-targeted therapy for PAC patients.

## Background

Pancreatic cancer (PAC) is one of the most aggressive and lethal malignancies. In data released by the American Cancer Society in 2019, PAC has a 5-year overall survival rate of only 9% and is one of the tumors with the lowest survival rate [1]. Despite the major advances in surgical techniques and adjuvant medical therapy, it is clear that the survival rate of PAC has not improved significantly. Meanwhile, more and more studies have shown that the high mortality and poor prognosis of PAC are closely correlated to changes in the TME [2]. Recently, bioinformatics-based therapy has become a promising tool in modern oncology. As such, it is pressing to apply to PAC and identify potential therapeutic targets.

Hypoxia is an inherent feature in the microenvironment of solid tumors, which can promote tumor survival and also lead to tumor proliferation and metabolism [3]. It is well-known that PAC possesses a complex TME that comprises myofibroblasts and immune cells [4]. Due to the strong association between hypoxia and fibrosis, PAC is also considered to be the most hypoxic tumor. The highly hypoxic environment produces a large extent of alterations in the tissue structure and cells of PAC in the TME [5]. For example, hypoxia is an important activator of pancreatic stellate cells (PSCs), which not only exacerbates the deterioration of the tumor hypoxic microenvironment [6] but also reduces the migration of natural killer (NK) cells and CD4+ and CD8+ T cells in the tumor stroma and accelerates the differentiation of myeloid-derived immunosuppressive cells to disrupt the balance of tumor immune microenvironment [7].

It has been found that hypoxia was served as a primary factor in the formation of tumor immunosuppressive microenvironment, which enhances tumor immune evasion by suppressing the anti-tumor immune responses [8]. Generally, hypoxia promotes the development of immunosuppressive cell populations (MDSC, Treg cells, M2-like macrophages, and immunosuppressive cytokines) in the microenvironment but also reduces the killing, survival, and migration of effector cells ( NK cells, CD4+ and CD8+ T cells), thereby impairing the regulation of anti-tumor immunity [9]. In recent years, many clinical trials have been performed on immunotherapy for tumors, but the efficacy of clinical treatment achieved on PAC are limited [10]. Although the specific mechanism of PAC immunotherapy resistance is still unclear, we believe that it is inseparable from the “barren soil” caused by hypoxia.

In the present study, we downloaded the mRNA profile data of PAC patients from the TCGA and GEO database and extracted genetic data related to hypoxia to construct a hypoxia risk model. We first conducted prognostic prediction for patients with PAC based on this risk model. On this basis, we further searched for the interrelation between hypoxia and tumor immune microenvironment. In the future, this approach will be crucial for researchers to develop new combination treatment strategies.

## Methods

### Data acquisition

Clinical information and RNA-sequencing expression date of 186 PAC patients were collected from the TCGA (http://cancergenome.nih.gov/) as a training set. Subsequently, the corresponding information of 112 PAC patients from the GSE78229 and GSE57495 datasets were downloaded from the GEO database as a validation set (https://www.ncbi.nlm.nih.gov/geo/). The two chip datasets in GEO were combined for batch normalization, and the combined result was used for further analysis. Tumor immune gene set and hallmark gene set were downloaded from TIP (http://biocc.hrbmu.edu.cn/TIP/) and GSEA (https://www.gsea-msigdb.org/gsea/index.jsp) respectively. All *P* values were subjected to multiple testing, taking an adjusted *P* value < 0.05 as a threshold.

### Construction of protein-protein interaction (PPI) network

String Database was used to construct the PPI network for downloaded hypoxia-related genes. R software language was utilized to analyze the correlation of hypoxia-related genes in the protein interaction relationship network, and then screened them as key core genes based on the number of interrelationships.

### Constitution of a risk model

First, we performed univariable Cox regression analysis on the hypoxia core genes to obtain prognostic-related hypoxia genes. They were then analyzed by multivariable Cox regression to obtain the genes for building the model and their coefficients. The risk score of each patient was calculated according to the obtained genes, and then patients were divided into low and high hypoxia risk groups based on the median risk score. The risk score formula was constructed as:

$$ \mathrm{Risk}\ \mathrm{score}={\sum}_{i=1}^{\mathrm{n}}\left({Exp}_i\ast {Coe}_i\right) $$

where *N* = 4, *Exp*_*i*_ indicated the expression level for each hypoxia genes, and the *Coe*_*i*_ indicated the corresponding multivariable Cox regression coefficient.

### Survival analysis

Overall survival (OS) analysis of PAC patients with low- and high- risk groups was performed by Kaplan-Meier method employing survminer and survival packages in R. Univariate and multivariate COX regression analysis was carried out to determine whether the risk score can be distinguished from other conventional clinical features as an independent prognostic factor for PAC patients. Bilateral *P* values less than 0.05 were considered statistically significant, and the hazard ratio (HR) was calculated for 95% confidence intervals. A ROC curve created by the survivalROC R package was used to evaluate the accuracy and reliability of risk model for predicting the patients’ OS.

To better assess the 1-, 3-, and 5-year survival probabilities of patients with PAC, we utilized the total independent prognostic factors to construct a nomogram.

### Gene set enrichment analysis (GSEA)

GSEA was carried out in the enrichment of the MSigDB Collection (h.all.v7.0.cymbols.gmt) to determine the regulation of signaling pathways in the hypoxia gene set between low and high hypoxia risk groups. Gene set permutations were conducted 1000 times for each analysis. *P* < 0.05 was considered statistically significant.

### Evaluation of immune cell type components

CIBERSORT is a tool for evaluating the proportion of various cell subtypes from a mixed cell samples through RNA-seq expression profiles and is the commonly cited method to estimate and analyze the immune cell infiltration [11]. We used CIBERSORT to evaluate the proportion of 22 immune cell subtypes in the high- and low-risk group, including CD8 T cells, resting memory CD4 T cells, and resting NK cells. The sum of the scores for the total immune cell types in a sample is equal to 1.

## Results

### Characterization of hypoxia risk score to predict PAC prognosis

**Fig. 1:**
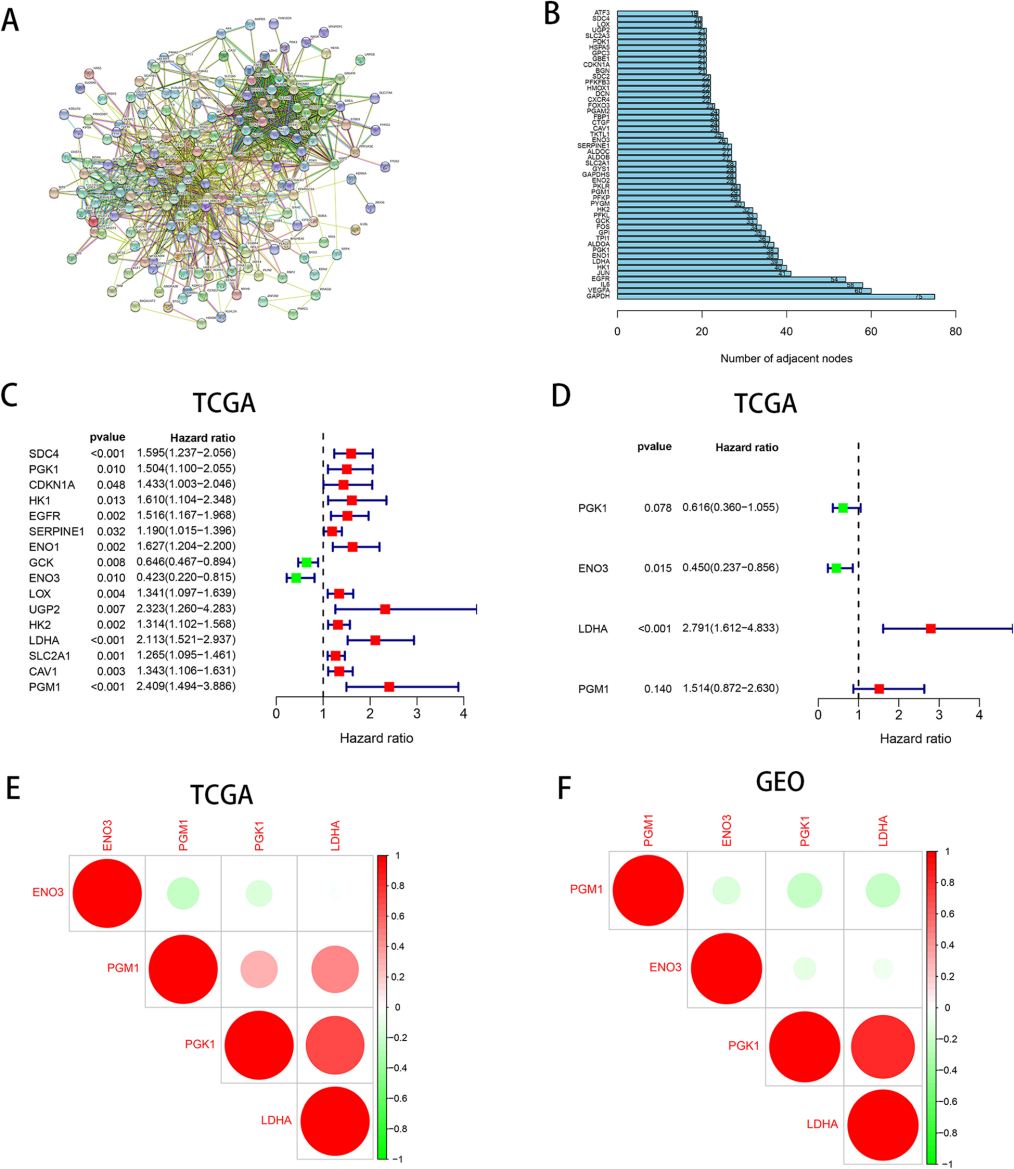
Characterization of hypoxia risk score to predict prognosis of PAC. **a** Protein–protein interactions among 200 hypoxia-associated genes. **b** The 50 genes with the highest interaction degrees were extracted. **c** Sixteen hypoxia genes associated with patient’ OS were confirmed by univariate Cox regression. **d** Four hypoxia-related genes were chosen to establish a hypoxia risk model by multivariate Cox regression. **e**, **f** Spearman correlation analysis of four hypoxia genes in the TCGA and GEO databases

We downloaded the hypoxia-associated gene set from the GSEA (hallmark-hypoxia), which contains more than 200 genes involved in hypoxia regulation pathways. To further understand the relationship between these hypoxia-related genes, we utilized the string online data (http://string-db.org) to conduct a network analysis of protein-protein interactions for these genes, and then extracted the most interconnected genes as core genes (Fig. 1a). Results showed that the 50 genes with the highest interaction levels were maintained, including GAPDH, VEGFA, IL6, EGFR, JUN, HK1, LDHA, ENO1, PGK1, ALDOA, indicating their crucial role in the hypoxia regulation process (Fig. 1b).

To build a hypoxia risk model that can predict the prognosis of PAC patients, we carried out univariate and multivariate Cox regression analysis on 50 hypoxia core genes in the TCGA training dataset (Tables 1 and 2). In the univariate Cox regression analysis, we confirmed 16 hypoxia genes associated with patient’ OS (Fig. 1c). In the following multivariate Cox regression analysis, the final 4 genes with a *P* value < 0.05 were chosen to establish the prognostic model, including PGK1, ENO3, LDHA, and PGM1 (Fig. 1d). The formula to calculate the risk score is defined as follows:

$$ \mathrm{Risk}\ \mathrm{score}=\left(-0.484\times \mathrm{PGK}1\right)+\left(-0.799\times \mathrm{ENO}3\right)+\left(1.026\times \mathrm{LDHA}\right)+\left(0.415\times \mathrm{PGM}1\right) $$

**Table 1:** A total of 16 hypoxia-related genes significantly associated with overall survival according to univariate Cox regression analysis. Hazard ratio (HR); low 95% confidence interval of hazard ratio (HR.95 L); high 95% confidence interval of hazard ratio (HR.95H)

Id	HR	HR.95L	HR.95H	*P*-value
HK1	1.610	1.104	2.348	0.013
SLC2A1	1.265	1.095	1.461	0.001
PGM1	2.409	1.494	3.886	0.0003
ENO1	1.627	1.204	2.200	0.002
SDC4	1.595	1.237	2.056	0.0003
PGK1	1.504	1.100	2.055	0.010
CAV1	1.343	1.106	1.631	0.003
SERPINE1	1.190	1.015	1.396	0.032
ENO3	0.423	0.220	0.815	0.010
GCK	0.646	0.467	0.894	0.008
LOX	1.341	1.097	1.639	0.004
CDKN1A	1.433	1.003	2.046	0.048
HK2	1.314	1.102	1.568	0.002
UGP2	2.323	1.260	4.283	0.007
EGFR	1.516	1.167	1.968	0.002
LDHA	2.113	1.521	2.937	0

**Table 2:** Details of the 4 hypoxia-related genes significantly associated with overall survival used to build the hypoxia risk model. Hazard ratio (HR); low 95% confidence interval of hazard ratio (HR.95 L); high 95% confidence interval of hazard ratio (HR.95H)

Id	HR	HR.95L	HR.95H	*P*-value
PGM1	1.514	0.872	2.630	0.140
PGK1	0.616	0.360	1.055	0.078
ENO3	0.450	0.237	0.856	0.015
LDHA	2.791	1.612	4.833	0.0002

There was no significant correlation among the four hypoxia-related genes selected by us (Fig. 1e, f).

### Prognostic efficacy of the hypoxia risk score in PAC patients

**Fig. 2:**
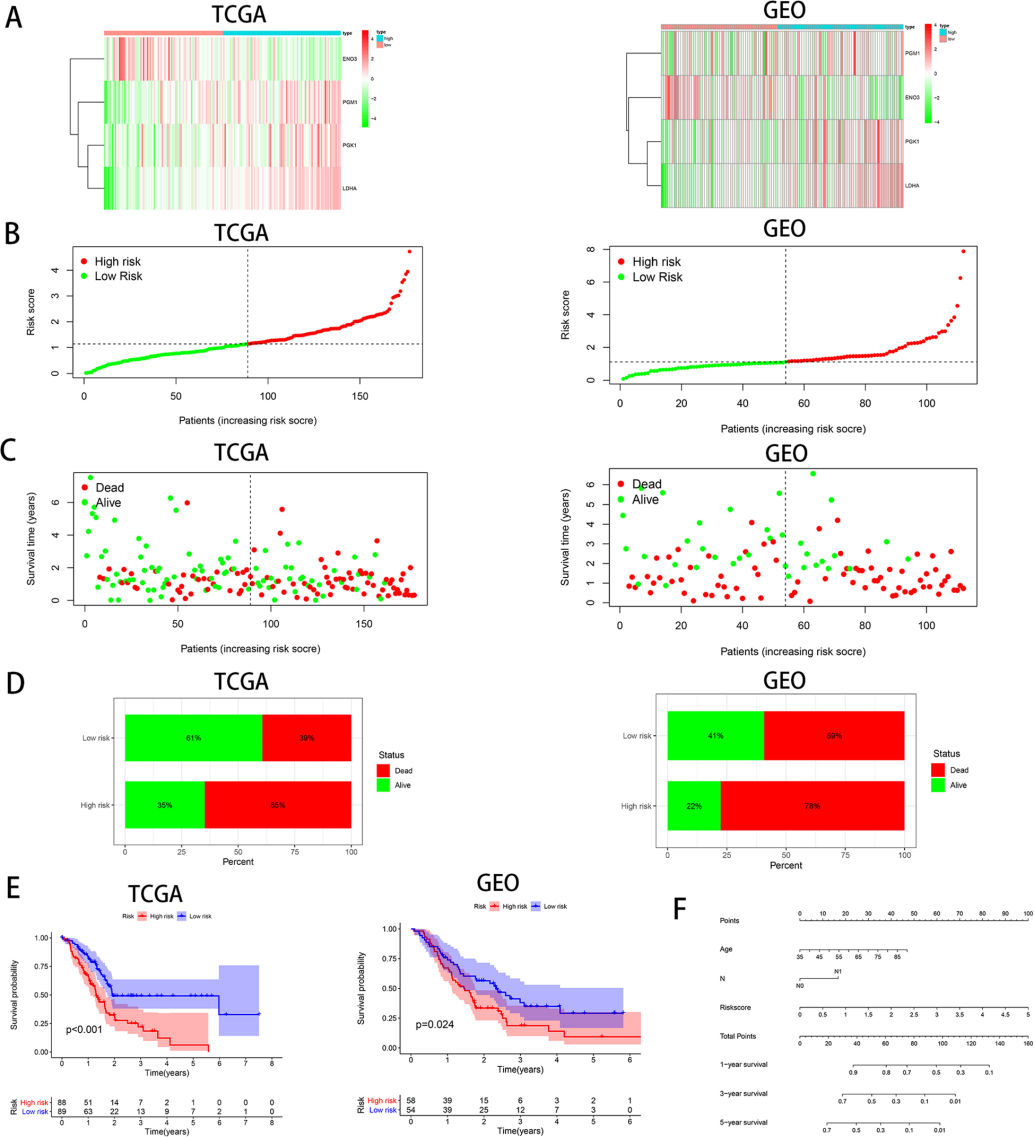
Prognostic efficacy of the hypoxia risk score in PAC. **a** The expression of four hypoxia-related genes in high and low hypoxia risk groups from the TCGA and GEO databases. **b** The relationship between hypoxia risk score and patient risk level. **c** Patient survival status distribution in the high and low hypoxia risk groups. The dot indicates the status of the patient ranked according to the increasing risk score. **d** The mortality rate of patients in high- and low- risk groups. **e** Kaplan-Meier overall survival analysis of PAC patients in high- and low-risk groups in the TCGA and GEO datasets. **f** Nomogram predicting the 1-, 3-, and 5-year survival probability of each individual patient

Studies have found that hypoxia can exacerbate the aggressiveness of tumors, so we further assessed the efficacy of hypoxia risk score on the prognosis of PAC. In the heatmap (Fig. 2a), we found that the expression of three hypoxia-associated genes in the high-risk group was significantly increased for both TCGA and GEO databases, indicating that PAC patients in the high-risk group may be more prone to form a hypoxic TME. Meanwhile, our research data found that hypoxia risk score was increased accompanying higher patient risk level (Fig. 2b), and the mortality rate of patients in the high-risk group was remarkably higher than that in the low-risk group (Fig. 2c, d). Besides, we used Kaplan-Meier analysis to estimate the prognostic value of hypoxia risk score in PAC patients. The results showed that high risk score was obviously associated with bad prognosis in the TCGA cohort, which was further validated from the GEO cohort (Fig. 2e).

Moreover, we created a nomogram to further calculate the survival probability of each patient more accurately. Set the scoring criteria according to the regression coefficient of all independent factors, and then the score value of each independent factor was given to calculate the total scores in each individual. The 1-, 3-, and 5-year survival probabilities of each individual was achieved via the function conversion relationship of total scores (Fig. 2f).

### Hypoxia risk score shows great feasibility for prognosis evaluation

**Fig. 3:**
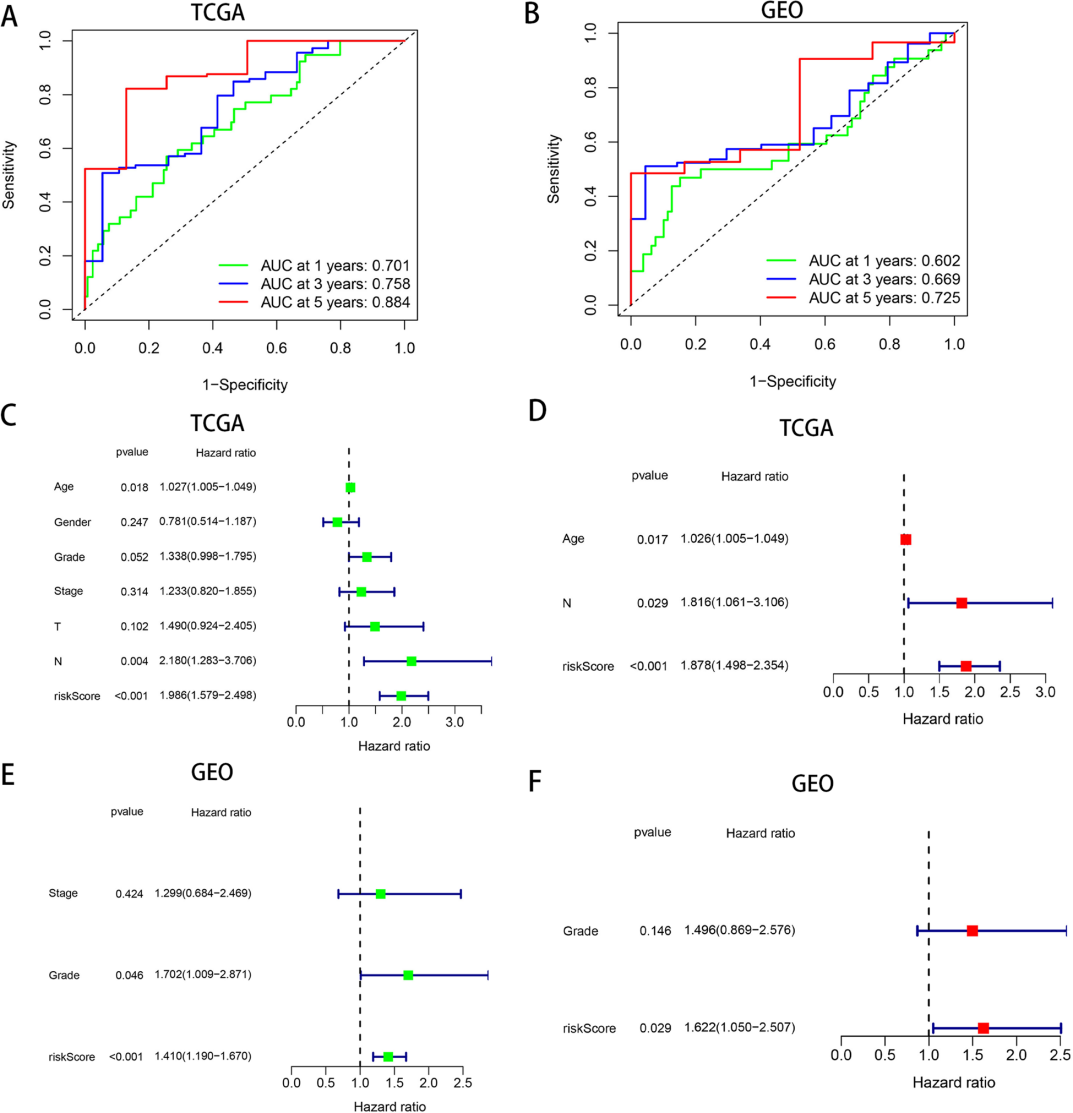
Prognostic value of the hypoxia risk score in PAC. **a**, **b** ROC curve showing the prognostic value of hypoxia risk scores in patients. **c**–**f** Univariate and multivariate Cox analyses evaluating the independent prognostic value of the hypoxia risk score in terms of OS in PAC patients

We carried out the received operating characteristic (ROC) curve to further assess the predictive accuracy and reliability of hypoxia risk score for the survival rate. In the TCGA cohort, the area under ROC curve (AUC) was 0.701 at 1 year, 0.758 at 3 years, and 0.884 at 5 years, respectively, demonstrating the predictive power of our risk model (Fig. 3a). We further validated this result in an independent cohort using GEO (Fig. 3b).

We applied univariate and multivariate Cox regression analysis to examine whether the hypoxic risk score could be an independent prognostic factor for predicting survival in patients with PAC. First, univariate Cox regression analysis showed that tumor grade, N stage, age, and hypoxia risk score were associated with OS in TCGA cohort (Fig. 3c). Then, multivariate Cox regression analysis indicated that hypoxia risk score was independently correlated with unfavorable overall survival of PAC patients, which could act as an independent prognostic factor in PAC (Fig. 3d). Similarly, validation result of the GEO cohort also demonstrated that hypoxia risk score can be used as an independent prognostic factor (Fig. 3e, f).

### GSEA identifies hypoxia signaling pathways

**Fig. 4:**
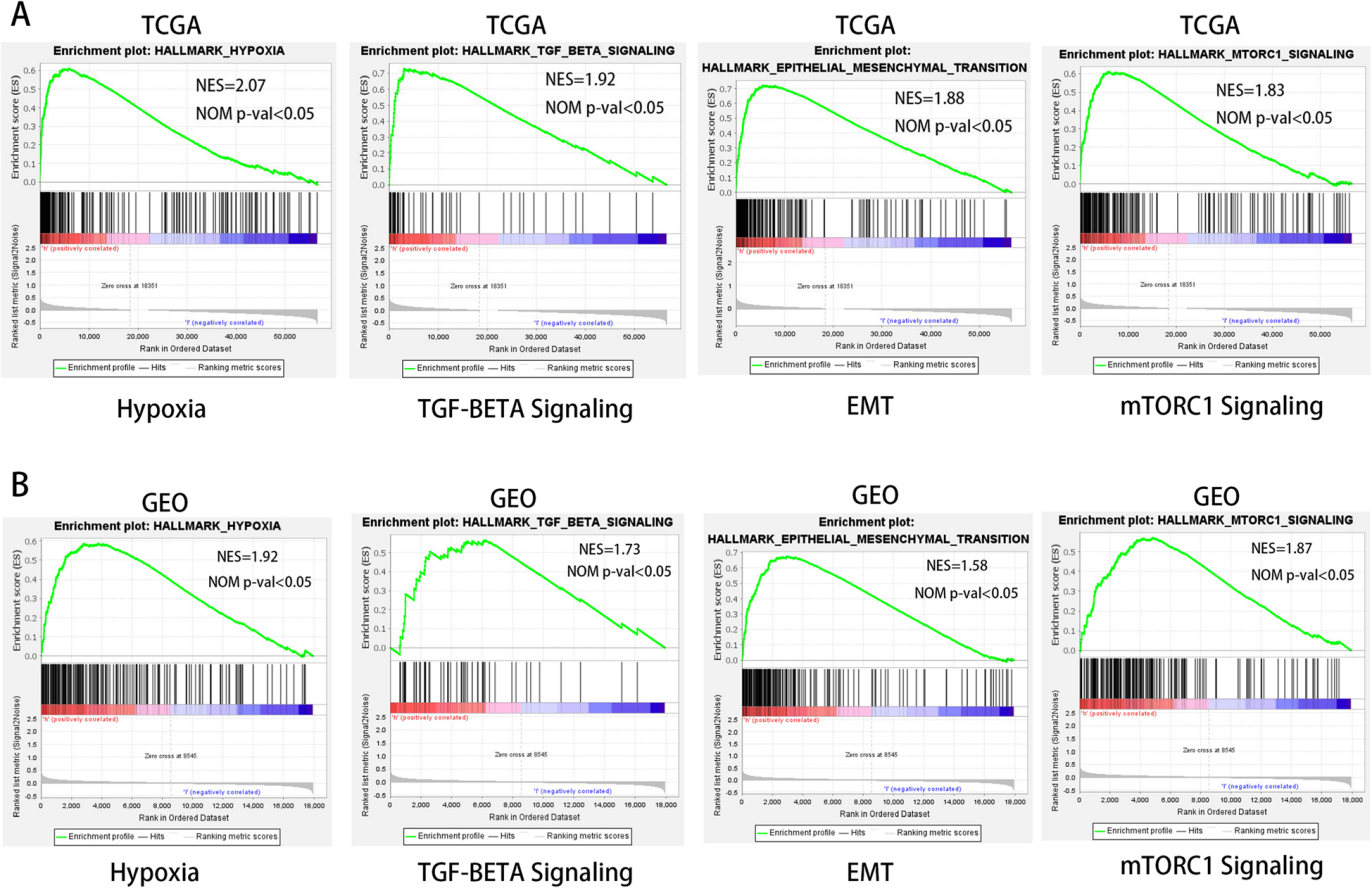
GSEA enrichment between high and low hypoxia risk groups. **a** GSEA indicating that genes in the high hypoxia risk group were enriched for the characteristics of malignant tumors. **b** This result was validated by GEO datasets. Normalized enrichment score (NES) > 1 and nominal *p* value (NOM *p*-val) < 0.05 were considered significant gene sets

We carried out the GSEA to further study signaling pathways activated by hypoxia-related genes in the high-risk group. The results showed that more genes in the high-risk group of the TCGA dataset were significantly enriched in multiple pathways such as hypoxia, TGF-β signaling, epithelial-mesenchymal transition, and mTORC1 signaling (Fig. 4a). They were all associated with the processes including tumor metastasis, proliferation, and anti-apoptosis. This result was validated by GEO datasets, which completely echoed the result (Fig. 4b).

### Analysis of immune cells in the high and low hypoxia risk group for PAC

Increasing studies indicated that hypoxia plays an integral role in tumor immune tolerance. It promotes the formation of the immunosuppressive microenvironment by means of abnormal immune cell activation, secretion of immunosuppressive factors, and downregulation of immune cells to protect tumor cells from immune cell attack.

**Fig. 5:**
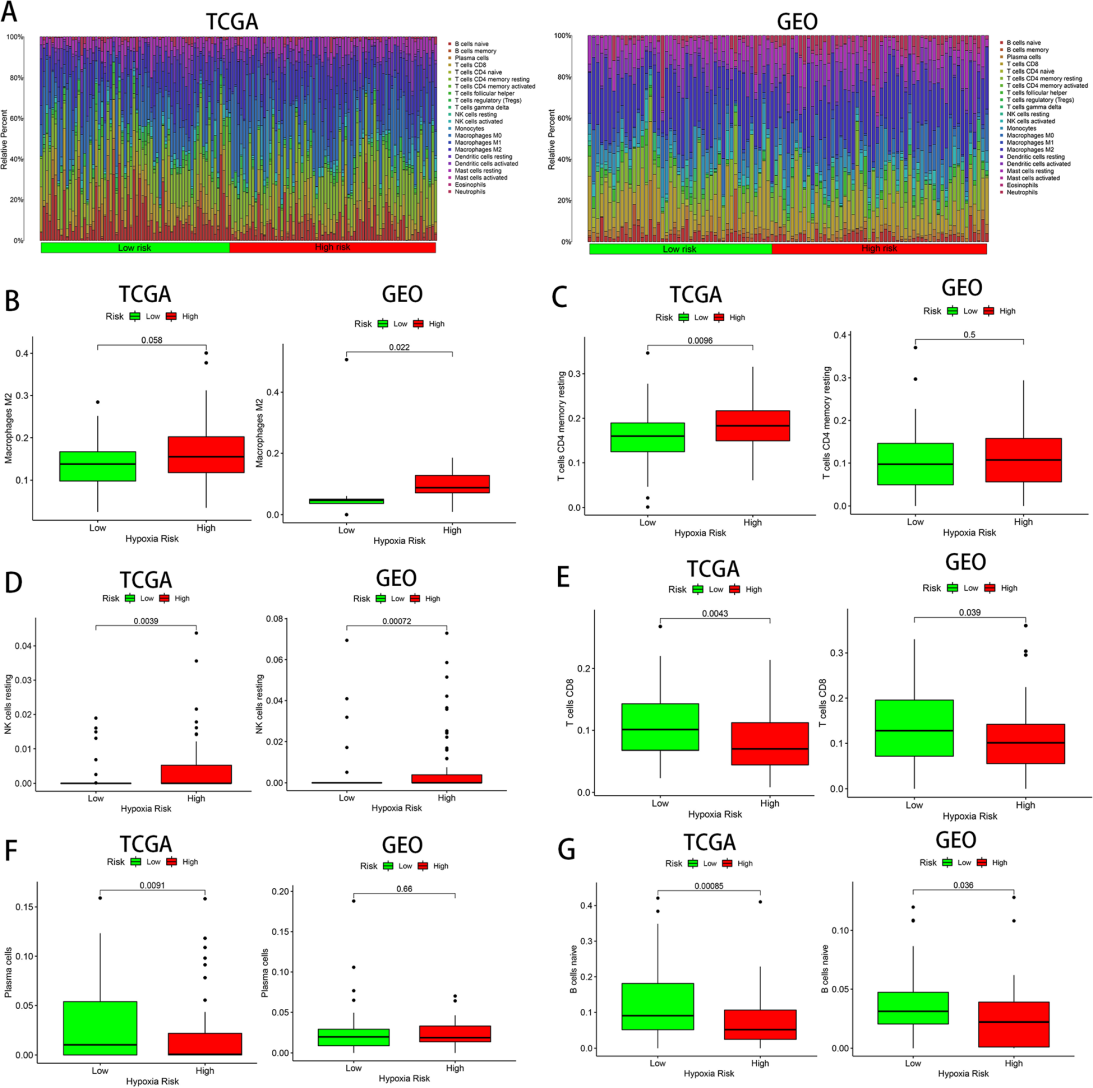
Analysis of immune cells in the high and low hypoxia risk group for PAC. **a** Relative proportion of immune infiltration in high and low hypoxia risk patients. **b**–**g** Box plot visualizing the differentially infiltrated immune cells in the low- and high- risk patients.

The CIBERSORT method was utilized to analyze the infiltration of 22 subpopulations of immune cells between the low- and high-risk groups of PAC patients. The results obtained from 186 PAC patients in TCGA and 112 patients in GEO were summarized in Fig. 5a. It was demonstrated that the proportion of immunosuppressive cells (M2 macrophages), resting memory CD4+ T cells, and resting NK cells (Fig. 5b–d) was significantly higher in the high hypoxia risk group. Besides, the levels of CD8 T cells, plasma cells, and naive B cells (Fig. 5e–g) were significantly reduced in PAC patients with high-risk score.

Thus, it can be seen that targeting hypoxia research is very important for future immunotherapy for tumor patients.

### High hypoxia risk tended to an immunosuppressive microenvironment

Tumor immunotherapy is a therapy that restores and enhances the host’s immune system to recognize and eradicate cancer. Immunotherapy involves a series of immune-related cell mediated tumor killing processes. Next, we examined the expression level of genes unfavorably regulating these processes in high- and low-risk groups. We then downloaded gene signatures on the Tracking Tumor Immunophenotype website (http://biocc.hrbmu.edu.cn/TIP/index.jsp) [12].

**Fig. 6:**
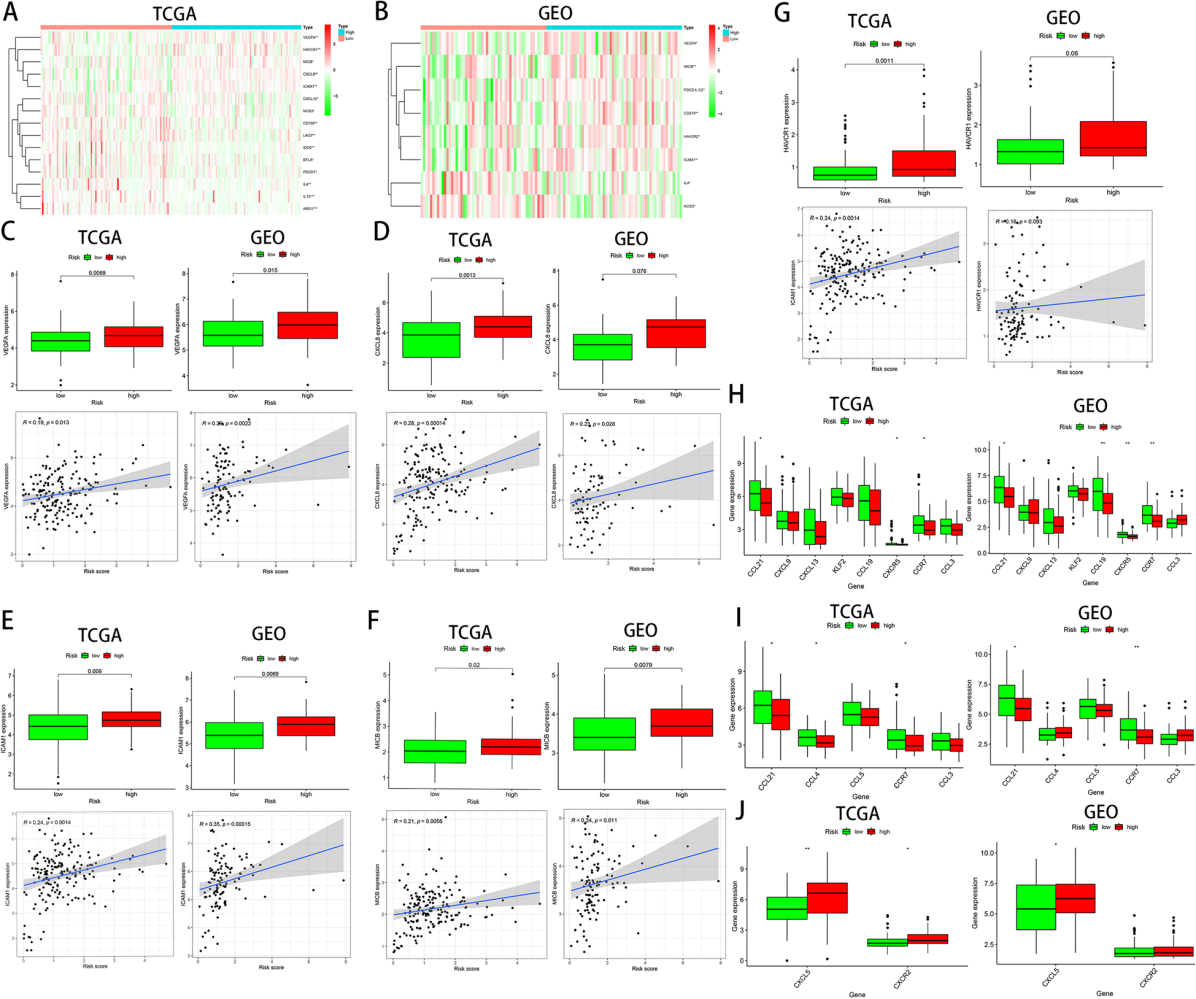
High hypoxia risk tended to an immunosuppressive microenvironment. **a**, **b** The heatmap displaying the expression of the gene set involved in the negative regulation of anti-tumor immunotherapy in the low and high hypoxia risk groups in the TCGA and GEO databases. **c**–**g** The expression levels of VEGFA, CXCL8, ICAM1, MICB, and HAVCR1 in high and low hypoxia risk groups. Correlation between the expression of VEGFA, CXCL8, ICAM1, MICB, and HAVCRI and hypoxia risk score. **h**–**j** The expression of genes that positively regulating T cells, dendritic cells, and MDSC cells in the low and high hypoxia risk groups.*P < 0.05, **P < 0.01, and ***P < 0.001

The results showed that the expressions of VEGFA, HAVCR1, CXCL8, MICB, and ICAM1, which were positively associated with hypoxia risk score, were significantly higher in the high-risk group (Fig. 6a–g), and the analysis found that most of them were related to the regulation of HIF-1α. For the present study, most of the immunosuppressive cytokines and immune checkpoints were not found to present a significant increasing trend in the high hypoxia risk group. We thought this might indicate that they are not the main drivers of the hypoxia-induced immunosuppressive microenvironment in PAC.

We further analyzed the expression of genes that positively regulating T cells, dendritic cells, and Myeloid-derived suppressor cells (MDSCs). As seen in Fig. 6h, i, the expression of regulatory genes of most anti-tumor immune effector cell was obviously downregulated in the high hypoxia risk group, including T cell regulatory genes CCL21, CXCR5, and CCR7 and dendritic cell regulatory genes CCL21, CCL4, and CCR7. Conversely, MDCS cell regulatory genes CXCL5 and CXCR2 were significantly upregulated in the high-risk group (Fig. 6j). This further confirms that the activity of effector cells in the immune microenvironment of PAC patients with high-risk score is reduced, while the immunosuppressive cell is increased.

## Discussion

Intratumoral hypoxia is a common characteristic of the TME, but also a prominent biological feature for PAC [3]. The hypoxic microenvironment of tumors is considered to be the main mechanism leading to tumor resistance to various treatments such as radiotherapy, chemotherapy, and immunotherapy [13, 14]. Immunotherapy refers to killing tumor cells by improving the antitumor immune responses of tumor patients, which is a promising tumor treatment method. Inhibition of immune checkpoint to enhance immune cell-mediated tumor killing has shown a promising result in many cancers (e.g., melanoma, colorectal cancer), but the effect in some solid tumors including PAC is disappointing [15, 16]. The existence of this phenomenon has been a subject of active debate. However, the potential effect of hypoxia as a key microenvironmental factor on the treatment of PAC is still being explored.

The risk model in our research was constructed by four hypoxia-related genes, and most of them were highly upregulated under hypoxic conditions. It was found that hypoxia-inducible factor 1/2α (HIF1/2α) can activate the expression of LDHA in PAC to achieve the purpose of promoting the proliferation and metastasis of PAC cells [17]. PGK1, a major enzyme in glycolysis, has been reported overexpressed in numerous malignancies. Studies have demonstrated that PGK1 is directly regulated by HIF-1α and acts as a promoter of metastasis in gastric cancer and colon cancer [18, 19]. Similarly, PGM1 was also shown to be upregulated under hypoxic environment [20]. In fact, hypoxia-associated signature for predicting the diagnosis, prognosis, and immune landscape has been used in a variety of tumors. For example, a hypoxia model developed by Zhang et al. could be regarded as a potential biomarker for diagnosis, prognosis, and recurrence of hepatocellular carcinoma [21]. And Mo et al. showed that the hypoxia-related gene signature could be considered as a prognostic factor and may guide the choice of immunotherapy in lung adenocarcinoma [22]. In this study, our hypoxic risk model composed of four genes can also be flexibly applied to PAC’s analysis.

Numerous studies have shown that hypoxia may induce tumor immune escape in a range of ways, including (A) reduce the activity of effector cells (NK cells, CD4+ and CD8+ T cells), (B) decrease the production and release of effector cytokines, (C) support the activity of immunosuppressive cells (Tregs, M2-like macrophage and MDSC), and (D) induce the expression of immunosuppressive cytokines.

The activation of NK cells and cytotoxic T lymphocytes (CTLs) is a crucial step in the tumor immune response. Accumulating evidence suggests that the low infiltration degree of NK cells, CD4+, and CD8+ T cells is a biomarker of poor prognosis and adverse clinical outcomes [23, 24]. However, it has been shown that hypoxia can suppress the activity of effector T cells and NK cells, which could result in decreased immune function. For example, hypoxia will enhance the process of glycolysis and glutamine decomposition, which will cause elevated levels of lactate production in the TME [25]. However, the acidic microenvironment not only inhibits the activity of T cells and the release of cytokines but also prevents NK cells from secreting TNF-α, IFN-y, perforin, and granzyme B [26, 27]. Our data illustrated that for patients in high-risk group, CD8+ T cells were decreased while resting NK cells and resting memory CD4+ T cells were increased, suggesting the formation of tumor immunosuppressive microenvironment.

In a recent study, three independent studies conducted by MD Anderson Cancer Center, INSERM, and Lund University all proposed that the appearance of B cells and tertiary lymphoid structures in tumor tissues is associated with better prognosis of patients undergoing immunotherapy [28–30]. The researchers speculate that B cells in the front line of anti-tumor may produce antibodies to effectively fight tumor cells and may also act by supporting CD8+ T cells [28]. Moreover, the study of Lee et al. [31] demonstrated that knockout of the HIF-1α gene in the PAC mouse model resulted in elevated expression levels of B cells chemokine, suggesting that hypoxia reduces the penetration of B cells in tumors, which is also consistent with our findings. In summary, although both anti-tumorigenic and pro-tumorigenic effects of B cells have been reported, it is still an indisputable fact that the result of hypoxia-induced B cell reduction in PAC patients may have a negative impact on the patient’s prognosis and immunotherapy.

TAMs originate from the response of macrophages in the blood to tumor signals and can be divided into M1 type and M2 type. Activation of the M2 phenotype has been proved to promote tumor angiogenesis and tumor cell metastasis through secretion of angiogenic factors (VEGF-A, IL-6, MMP) and immune suppressive factors (IL-10, TGF-β) [32]. Previous studies have shown that hypoxia develops a functionally immunosuppressive microenvironment by stimulating the differentiation of macrophages into M2 type [33]. In this study, we observed a significantly higher proportion of the M2-like phenotype in the high hypoxia risk group, suggesting that this hypoxia risk model has the ability to predict the immune microenvironment.

Furthermore, we analyzed the genes that were significantly increased in the high hypoxia risk group, including VEGFA, CXCL8, HAVCR1, MICB, and ICAM1, and found that they were all related to the regulation of HIF-1α [34–37]. Studies have found that VEGFA affects the response of effector T cells and the development of lymphocytes by promoting suppressive immune cell populations, which is closely related to hypoxia and immunosuppressive TME [38–40]. Meanwhile, Chao et al. [41] found that CXCL8 inhibits the function of CD8+ T cells via increasing the expression of PD-L1 on the surface of macrophages to participate in the formation of the immunosuppressive microenvironment of gastric cancer. However, the immune-related regulatory role of CXCL8 in PAC is unclear. In addition, ICAM-1 and HAVCR-1 are both overexpressed in various cancers and are related to the malignant potential of tumor cells [36, 42]. It is known that HIF-1 is an important regulator of the TME. Due to the specific hypoxic TME of pancreatic cancer, the expression of HIF-1α in PAC tissue is higher than that of other solid tumors [43, 44]. Therefore, HIF-1α seems to be implicated in the construction of an immunosuppressive microenvironment under hypoxia in PAC.

Recently, increasing studies observed that immune checkpoint plays a crucial part in tumor immune evasion and the formation of tumor immunosuppressive microenvironment [45]. Indeed, immune checkpoint blockade therapy has achieved remarkable results in some tumors but has little effect on PAC. Thus, it was proposed that PD1, PDL1, and CTLA4 are likely not the primary immune checkpoint molecules involved in immune suppression of PAC [5]. Consistent with this evidence, our data showed that associated immune checkpoint molecules were not significantly higher in the high hypoxia risk group. Taken together, we speculate that immune checkpoint may not be the major factor contributing to the immunosuppressive microenvironment in PAC under hypoxic conditions.

In this study, we successfully establish a 4-gene-based hypoxia risk model that effectively predicts the prognoses of patients with PAC. Here, our model served as an independent prognostic factor for PAC patients and describes how hypoxia status disrupts the immune microenvironment in PAC. However, it is undeniable that the interaction between hypoxia and the tumor’s immune microenvironment is complex, and more independent cohorts and functional experiments are needed to conduct a deeper discussion. We still believe this study will provide insights into the potential value of hypoxia-targeted therapies and help to design unconventional combinatorial methods to enhance the efficacy of PAC therapies.

## Conclusions

By combining bioinformatics tools and related algorithms, we established and validated a hypoxia risk model to predict the prognosis of patients with pancreatic cancer and explored the changes in the tumor immune microenvironment under hypoxic conditions. This study found that patients with high hypoxia risk are associated with poor prognosis and the formation of an immunosuppressive microenvironment, which could bring new insights to tumor treatment.

## Data Availability

The data used to support the findings of this study are included within the article.

## Abbreviations

PAC: Pancreatic cancer
TME: Tumor microenvironment
OS: Overall survival
ROC: Received operating characteristic
CTLs: Cytotoxic T lymphocytes
GSEA: Gene set enrichment analysis
*P*: *P* value
HR: Hazard ratio
MDSCs: Myeloid-derived suppressor cells
GEO: Gene Expression Omnibus
TCGA: The Cancer Genome Atlas
HIF1/2α: Hypoxia-inducible factor 1/2α
